# Supplementary material for: Improved HPLC Conditions to Determine Eumelanin and Pheomelanin Contents in Biological Samples Using an Ion Pair Reagent
Source: Int J Mol Sci. 2020 Jul 20;21(14):5134. doi: 10.3390/ijms21145134 (PMC7404343; doi:10.3390/ijms21145134)
Supplement: Supplementary file 1 [file ijms-21-05134-s001.pdf]

Supplemental Information

Improved HPLC Conditions to Determine Eumelanin and Pheomelanin Contents in Biological Samples Using an Ion Pair Reagent

Shosuke Ito, Sandra Del Bino, Tomohisa Hirobe, Kazumasa Wakamatsu

Table S1. Comparison of levels of melanin markers in synthetic melanins between the improved HPLC method and the original HPLC method (n = 12)

	**AHPO (μg/mg)[a]**				
**Type of Melanin**	**PTCA**	**PDCA**	**PTeCA**	**TTCA**	**TDCA**
Dopa-M, native	5.37 / 4.96	7.17 / 7.27	1.84 / 1.25	0.00 / 0.13	0.00 / 0.09
Dopa+Cys (1:0.5)-M, native	5.24 / 4.04	4.22 / 4.27	0.95 / 0.70	5.76 / 6.55	3.55 / 3.80
Dopa+Cys (1:1)-M, native	4.34 / 3.83	1.17 / 1.03	0.24 / 0.17	5.52 / 6.82	2.07 / 2.30
DHI+DHICA (1:0)-M, native	2.38 / 2.32	1.81 / 3.04	4.40 / 3.82	0.00 / 0.00	0.00 / 0.00
DHI+DHICA (1:1)-M, native	41.7 / 39.8	1.35 / 2.08	2.84 / 2.14	0.00 / 0.00	0.00 / 0.00
DHI+DHICA (0:1)-M, native	129 / 123	1.76 / 2.52	0.95 / 1.00	0.00 / 0.00	0.00 / 0.00
Dopa-M, heated	7.50 / 6.89	6.43 / 6.87	2.95 / 2.03	0.00 / 0.31	0.00 / 0.13
Dopa+Cys (1:0.5)-M, heated	2.57/ 2.37	3.14 / 3.27	1.30 / 0.90	10.75/ 12.31	8.79 / 8.78
Dopa+Cys (1:1)-M, heated	2.49 / 2.38	0.90 / 0.97	0.24 / 0.46	11.65 / 13.43	6.54 / 6.88
DHI+DHICA (1:0)-M, heated	2.90 / 2.87	1.30 / 3.56	8.01 / 7.59	0.00 / 0.00	0.00 / 0.00
DHI+DHICA (1:1)-M, heated	8.93 / 8.21	0.93 / 1.95	4.71 / 4.26	0.00 / 0.00	0.00 / 0.00
DHI+DHICA (0:1)-M, heated	12.7 / 11.5	0.73 / 1.49	4.80 / 4.52	0.00 / 0.00	0.00 / 0.00
Averages	18.8 / 17.7 (n = 12)	2.58 / 3.19 (n = 12)	2.77 / 2.40 (n = 12)	5.61 / 6.50 (n = 4)	3.49 / 3.66 (n = 6)

[a] Values are reported for the improved HPLC method / the original HPLC method. For example, 5.37 PTCA is the value with the improved HPLC method while 4.96 is with the original HPLC method.

Table S2. Comparison of levels of melanin markers in mouse hair samples between the improved HPLC method and the original HPLC method (n = 10).

	AHPO (ng/mg)[a]			
**Coat color phenotype**	**PTCA**	**PDCA**	**PTeCA**	**TTCA**
Black	2828 / 2897	25.2 / 40.7	154 / 160	27.8/ 17.8
Black	3406 / 3397	30.0 / 27.1	194 / 186	27.8 / 25.4
Brown	787 / 812	17.6 / 23.3	34.1 / 29.3	23.2 / 20.4
Brown	877 / 854	22.9 / 16.1	47.5 / 29.3	23.2 / 42.6
Slaty	389 / 399	81.7 / 88.5	41.3 / 53.3	37.1 / 30.7
Slaty	525 / 514	107 / 103	63.2 / 67.3	51.0 / 47.4
Pink-eyed dilution	144 / 150	8.1 / 4.2	12.4 / 8.0	18.5 / 20.4
Pink-eyed dilution	189 / 182	7.5 / 5.2	15.4 / 10.7	13.9 / 18.9
Recessive yellow	129 / 127	30.0 / 32.9	10.3 / 5.3	111 / 102
Recessive yellow	149 / 153	32.7 / 20.8	16.5 / 5.3	125 / 130
Averages	942 / 949	36.3 / 36.2	45.9 / 45.6	58.8 / 55.5

[a] Values are reported for the improved HPLC method / the original HPLC method. For example, 2828 PTCA is the value with the improved method while 2897 is with the original method.

Table S3. Comparison of levels of melanin markers in human hair samples between the improved HPLC method and the original HPLC method (n = 12)

	AHPO (ng/mg)[a]				
**Hair color**	**PTCA**	**PDCA**	**PTeCA**	**TTCA**	**TDCA**
Black	178 / 187	18.6 / 21.8	48.1 / 49.1	33.8/ 38.2	17.3 / 21.2
Black	203 / 22.6	20.4 / 22.6	55.5 / 55.8	41.5 / 41.9	18.5 /20.1
Dark brown	141 / 175	10.8 / 14.7	51.1 / 52.1	27.4 / 40.6	13.6 / 15.5
Dark brown	188 / 210	17.1 / 20.3	62.2 / 61.4	34.0 / 45.0	17.6 / 18.8
Brown	110 / 97.0	8.8 / 7.3	33.3 / 46.9	20.3 / 22.3	12.2 / 10.3
Brown	123 / 128	9.8 / 7.0	43.1 / 46.5	25.0 / 37.8	13.6 / 18.4
Light brown	55.2 /54.2	3.9 /4.1	14.8 / 22.3	14.1 / 16.8	9.5 / 8.0
Light brown	50.0 / 50.7	5.0 / 3.5	14.8 / 22.3	17.8 / 19.8	6.8 / 8.0
Blond	8.4 / 9.2	2.0 / 1.2	6.2 / 17.4	8.1 / 10.0	4.1 / 4.6
Blond	6.8 / 6.1	1.5 / 1.2	4.9 / 11.2	6.1 / 5.7	2.7 / 2.3
Red	32.7 / 48.2	6.6 / 4.7	9.9 / 14.9	96.8 / 81.0	21.5 / 19.0
Red	21.9 / 24.2	7.2 / 7.5	7.4 / 14.9	144 / 143	21.6 / 18.9
Averages	93.2 / 99.6	9.3 / 9.7	29.3 / 34.6	39.1 / 41.8	13.3 / 13.8

[a] Values are reported for the improved HPLC method / the original HPLC method. For example, 178 PTCA is the value with the improved method while 187 is with the original method.

Table S4. Comparison of levels of melanin markers in human epidermal samples between the improved HPLC method and the original HPLC method (n = 18)

		Soluene-350 solubilization		AHPO (ng/mg)[a]					HI hydrolysis	
**ITA**	**Skin color typology**	**A500 (/mg)**	**A650 (/mg)**	**PTCA**	**PDCA[b]**	**PTeCA**	**TTCA**	**TDCA**	**4-AHP**	**3-AHP**
57.92	Very light	0.004	0.001	40.7 / 43,0	0.4 / 3.3	1.2 / 3.8	6.5 / 6.9	3.2 / 4.9	3.3	3.1
55.72	Very light	0.006	0.001	20.9 / 28,2	0.4 / 3.8	0.6 / 5.3	7.4 / 11.3	3.2 / 2.4	4.9	4.3
55.05	Very light	0.007	0.001	15.8 / 18.9	0.4 / 3.8	0.6 / 4.2	9.3 / 8.8	3.2 /4/9	4.3	2.8
54.08	Light	0.010	0.002	23.9 / 23.9	0.4 / 2.7	1.2 / 3.8	9.3 / 10.1	3.2 /6.1	4.8	2.9
47.6	Light	0.018	0.006	24.4 / 27.2	0.4 / 5.4	1.8 / 4.5	10.2 / 11.3	3.2 / 9.1	6.6	3.7
41.5	Light	0.026	0.006	74.3 / 86.3	1.8 / 6.0	4.7 / 4.5	20.4 / 22.7	6.3 / 10.3	15.2	6.3
36.03	Intermediate	0.017	0.004	62.1 / 65.1	1.6 / 3.3	4.1 / 4.5	18.5 / 16.6	3.8 / 7.3	14.1	5.8
32.82	Intermediate	0.026	0.005	78.5 / 84.6	2.0 / 7.6	3.0 / 3.8	27.8 / 31.5	5.1 / 9.7	15.7	7.3
29.56	Intermediate	0.020	0.004	55.1 / 59.8	1.3 / 6.5	3.6 / 8.3	18.5 / 15.2	7.6 / 8.5	19.5	7.1
19.21	Tan	0.036	0.008	134 / 140	3.8 / 7.6	5.3 / 6.1	48.1 / 47.9	13.9 / 15.8	38.2	13.6
19.38	Tan	0.037	0.008	93.8 / 95.8	3.4 / 4.4	5.3 / 7.6	42.6 / 41.6	12.7 / 12.2	54.1	18.3
19.12	Tan	0.026	0.005	96.5 / 98.2	1.3 / 5.4	4.1 / 13.6	27.8 / 23.9	10.1 / 9.7	21.2	6.6
-9.96	Brown	0.065	0.014	194 / 197	6.1 / 8.7	13.0 / 16.6	59.2 / 60.9	13.9 / 18.3	43.5	13.6
-0.59	Brown	0.085	0.018	241 / 241	8.3 / 10.9	17.8 / 18.2	72.2 / 74.0	15.2 / 17.9	45.5	14.7
-26.94	Brown	0.079	0.017	215 / 216	7.2 / 8.7	17.8 / 24.2	61.1 / 59.5	20.3 / 20.9	21.8	7.9
-49.5	Dark	0.100	0.022	288 / 284	7.8 / 9.8	23.1 / 31.0	70.3 / 60.9	17.7 / 17.0	26.3	10.0
-50.69	Dark	0.147	0.032	389 / 388	13.0 / 14.1	27.3 / 34.8	92.2 / 82.7	27.8 / 26.2	27.8	9.4
-70.85	Dark	0.171	0.037	533 / 500	12.3 / 13.1	35.6 / 39.0	92.1 / 92.0	30.4 / 29.2	36.4	13.1
Averages		0.0489	0.0106	143/144	3.9/7.0	9.5/13.0	38.5/37.7	12.5/12.8	22.4	8.4

[a] Values by the improved method/original method. For example, 40.7 PTCA is the value with the improved method while 43.0 is with the original method.

[b] An interfering peak with the original method affected the values.

Figure S1. Correlations among various melanin markers in human epidermis. The units on the x- and y-axes are 1/mg for A500 and A650 and ng/mg for the other markers.

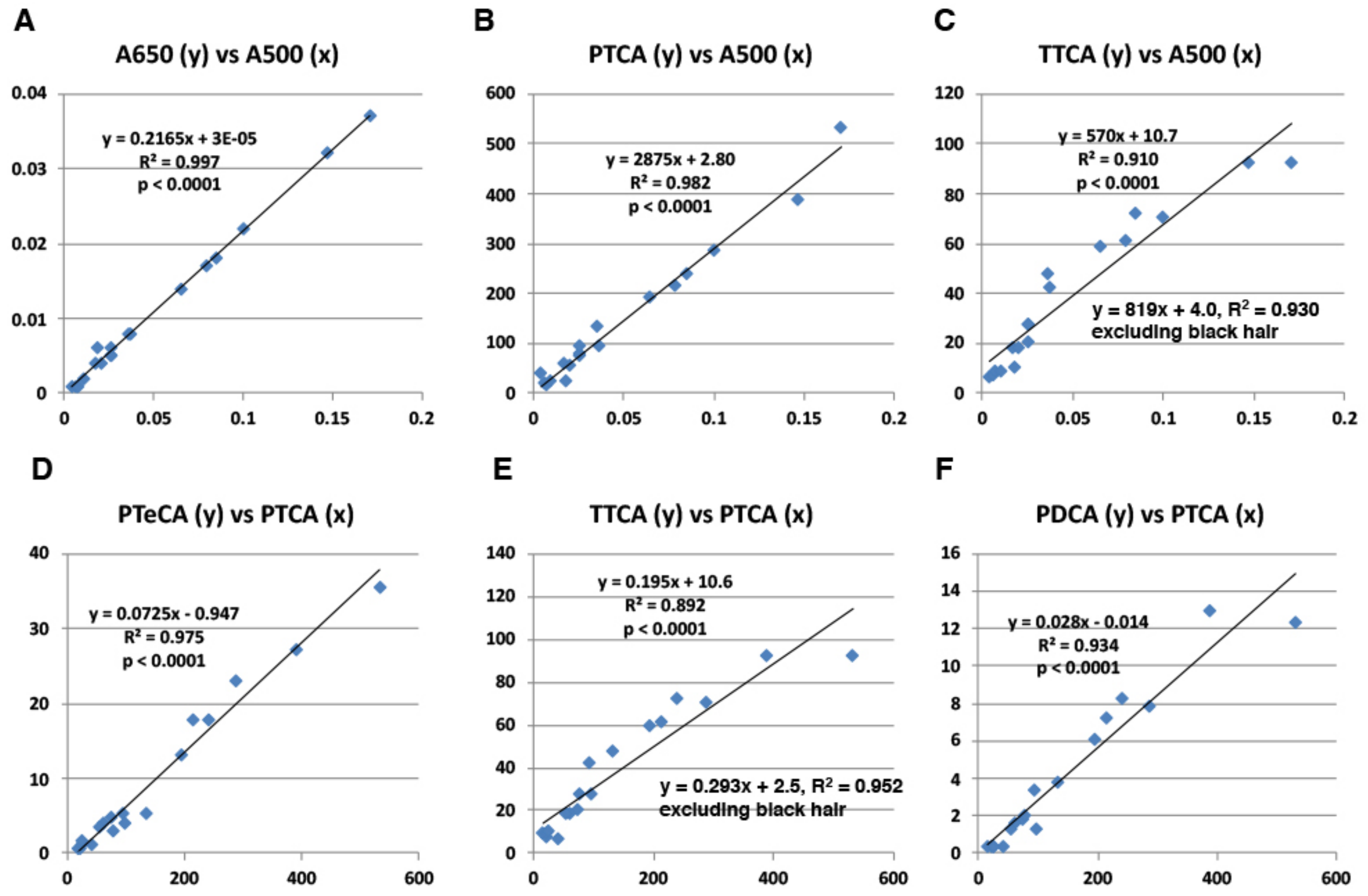

Figure S2. Correlation of melanin markers with ITA. The units for the y-axis is ng/mg.

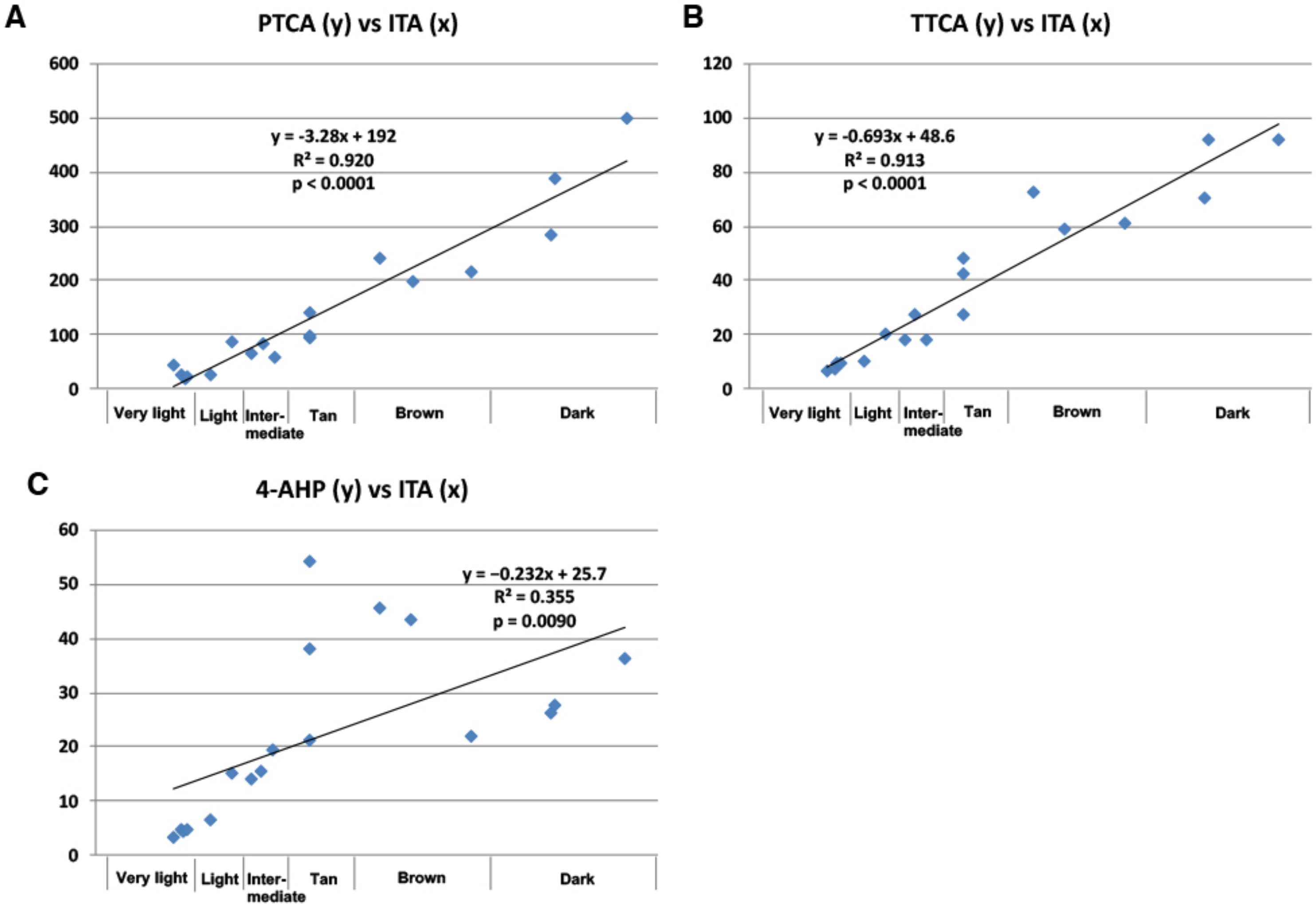

Figure S3. Microspecies distribution at various pH and strongest acidic pKa in PDCA, TDCA, PTCA, TTCA, and PTeCA.

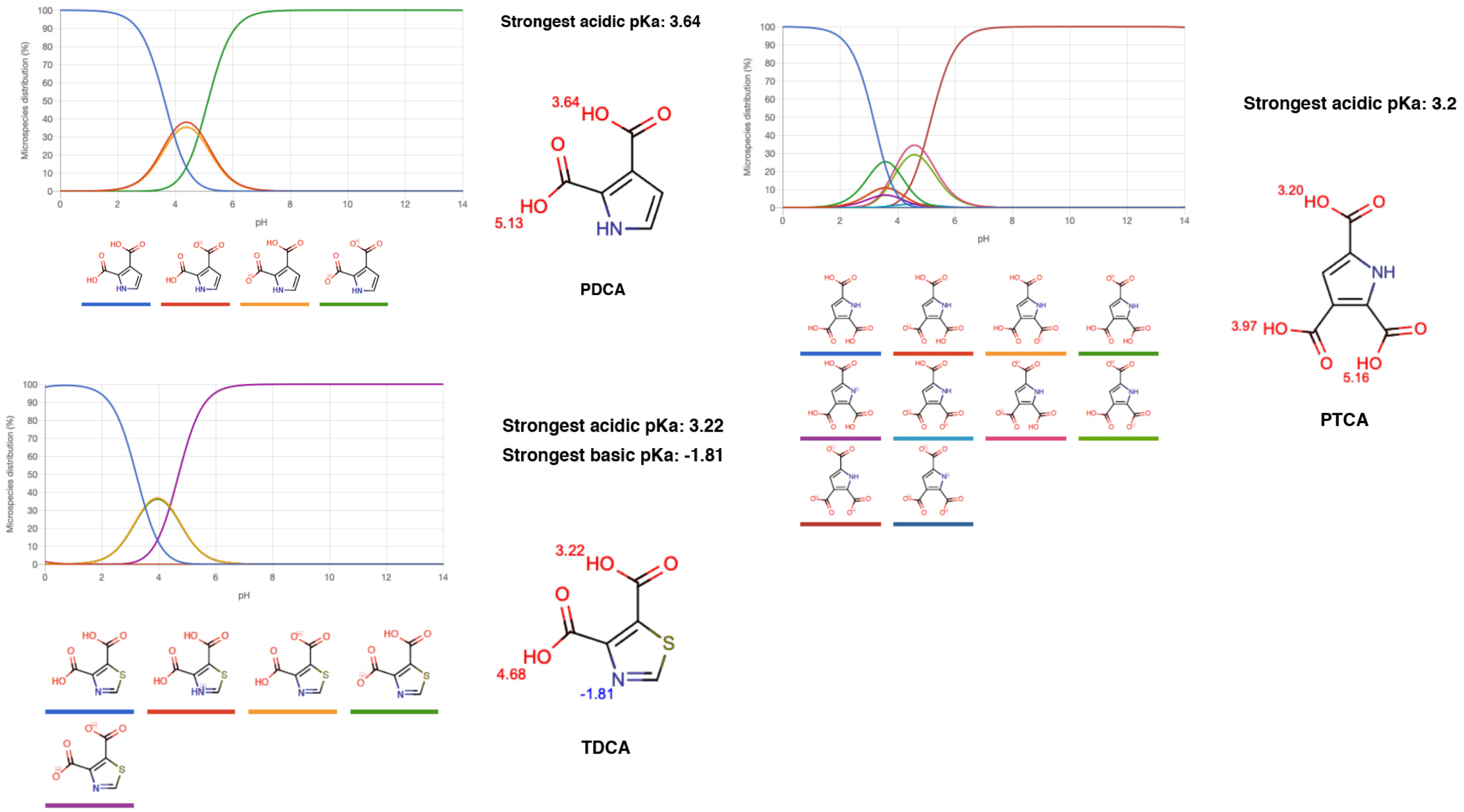

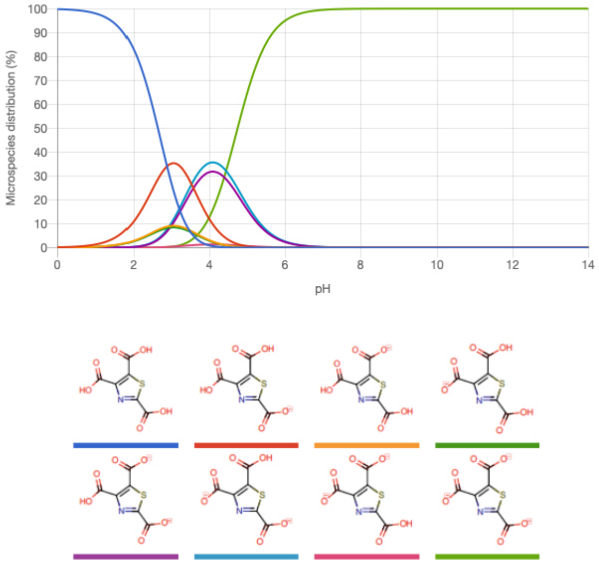

Strongest acidic pKa: 2.7

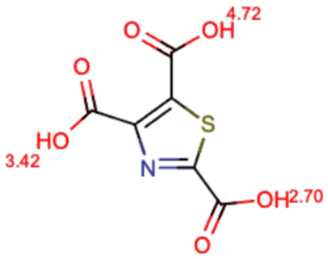

TTCA

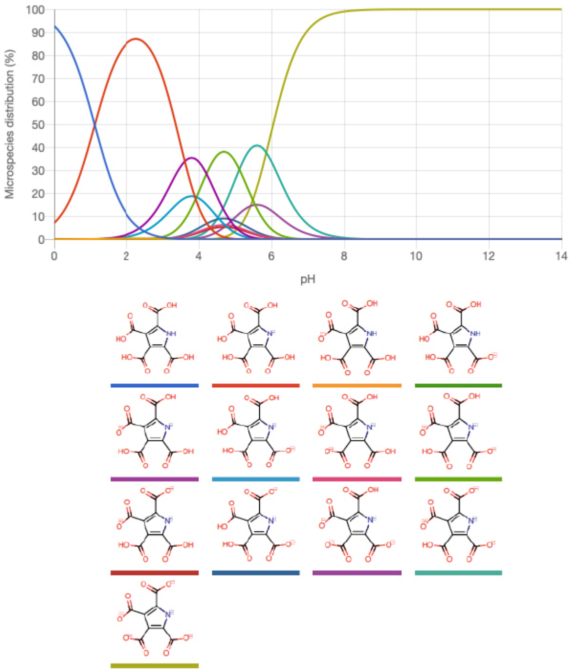

Strongest acidic pKa: 1.11

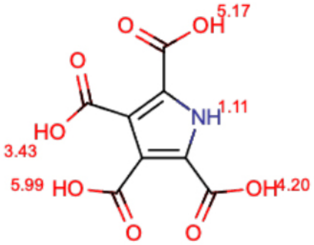

PTeCA
